# The effectiveness of mindfulness-based stress reduction (MBSR) on depression, PTSD, and mindfulness among military veterans: A systematic review and meta-analysis

**DOI:** 10.1177/20551029241302969

**Published:** 2024-11-21

**Authors:** Wendy Wen Li, Jaime Nannestad, Timothy Leow, Carolyn Heward

**Affiliations:** 1 8001 James Cook University, Australia; 2 Townsville University Hospital, Australia

**Keywords:** defence force, depression, MBSR, mental health, posttraumatic stress disorder (PTSD)

## Abstract

Thirteen studies were included in the current systematic review and meta-analysis with 1131 participants. Both within- and between-group comparisons demonstrated reductions in depressive and PTSD symptoms with medium effect sizes post MBSR intervention. Additionally, MBSR demonstrated small effects in improving mindfulness in veterans at post-intervention. Maintenance of treatment effects were observed at follow-up for the three outcomes during within-group comparisons. Treatment effects were maintained at follow-up between-groups for depression and mindfulness, but not for PTSD symptoms. Furthermore, there were no significant differences between MBSR and cognitive behavioural therapy/person-centred group therapy intervention groups in the three outcomes.

## Introduction

Military service places individuals in extreme environments and exposes them to traumatic combat scenes and potentially debilitating injuries (Haagen et al., 2015; Inoue et al., 2023). The consequences of this accumulation of trauma can impact mental health long after service personnel have left the armed forces (van Hooff et al., 2019). As a result, veterans face a heightened risk of mental health disorders; posttraumatic stress disorder (PTSD) and depression are the most prevalent mental health conditions that veterans face (Inoue et al., 2023) and they form the central focus of the current study. PTSD refers to a psychological disorder that develops after witnessing or experiencing a traumatic event(s) or circumstance(s) and includes intrusion symptoms relating to the trauma, avoidance of reminders of the traumatic event, negative alterations in thinking and mood, and alterations in arousal and reactivity (American Psychiatric Association, 2023). Depression is a mental health condition that negatively affects people’s mood, thinking, and behaviour, often resulting in prolonged periods of low mood or a loss of interest or pleasure in activities (WHO, 2023). Approximately 50% of Australian Defence Force (ADF) veterans have been diagnosed with a mental disorder, including PTSD and depression, with the prevalence of PTSD symptoms in veterans (17.7%) twice as high as in the general population (8.7%) (van Hooff et al., 2019). In the UK, the prevalence of PTSD symptoms in veterans (9%) is more than twice as high as that of active defence members (4%) (Stevelink et al., 2018).

Treatments recommended for military-related PTSD include trauma-focused therapies such as prolonged exposure therapy, cognitive processing therapy, and trauma-focused cognitive behavioural therapy (Haagen et al., 2015; Meis et al., 2021; Watkins et al., 2018). However, these treatments present various challenges. Firstly, the high noncompletion or “dropout” rates, ranging from 25–48% pose a significant challenge when treating PTSD (Sciarrino et al., 2022; Steenkamp et al., 2020), and recent reviews suggested that noncompletion rates for PTSD treatment tend to be higher in veteran populations compared to civilians (Edwards-Stewart et al., 2021; Varker et al., 2021). A review by Edwards-Stewart et al. (2021) found that the therapy attrition rate of veterans participating in trauma-focused therapies (24.3%) is higher than that of veterans participating in non-trauma-focused therapies (16.1%). Secondly, clinically significant symptom improvement in trauma-focused therapies is found to be varied among military veterans (Steenkamp et al., 2020). For example, in a sample of 960 veterans, Murphy and Smith (2018) found that 27.5% of participants had poor treatment response and experienced higher rates of depression, anxiety, and anger after a trauma-focused cognitive behavioural therapy intervention. Moreover, the participants’ PTSD symptoms were maintained at a 12-month follow up. Thirdly, benefits from prolonged exposure and cognitive processing therapies are found to be minor; compared to non-trauma focused treatments (Steenkamp et al., 2020). For instance, in a random controlled trial (RCT) comparing the effectiveness of transcendental meditation to prolonged exposure for PTSD treatment for veterans, 61% of participants who were treated with transcendental meditation showed clinically significant improvement compared to 41% treated with prolonged exposure (Nidich et al., 2018). Given these challenges, there is a growing need for alternative therapeutic approaches for veterans dealing with mental health disorders (Hundt et al., 2020).

Mindfulness-based intervention (MBI) is one such promising alternative therapy. Mindfulness is defined as “the awareness that emerges through paying attention on purpose, in the present moment, and nonjudgmentally to the unfolding of experience moment by moment” (Kabat-Zinn, 2013: 145). Mindfulness can be cultivated through meditation, a state of detached observation and awareness of the present moment (Kabat-Zinn, 2003), aiming to increase awareness of thoughts and feelings of the present moment and attend to the thoughts and feelings without judgement (Kabat-Zinn, 2003, 2013; Li et al., 2023b, 2024a; Omidi et al., 2013; Schure et al., 2018). Meditation practices encourage emotional regulation by enabling an individual to respond rather than react to stressful situations (Bishop, 2002; Li et al., 2023a). Mindfulness-based stress intervention (MBSR) is one of the most widely studied MBIs. MBSR is a non-trauma focused treatment that is delivered through an eight-week standardised group program (Kabat-Zinn, 2003) and employs a mind-body connection and a relaxed non-judgemental state of mind to aid in reducing PTSD symptoms, arousal, and improve mood (Kim et al., 2013). A meta-analysis of randomised controlled trials (RCTs) in a general PTSD population, revealed a significant medium effect of MBSR on reducing PTSD symptoms (Hedges’ *g* = 0.46, *p* < 0.001) when compared to the treatment as usual (TAU; Liu et al., 2022). A meta-analysis of the effect of MBSR on the mental health of breast cancer survivors found a medium effect on reducing depression symptoms (Cohen’s *d* = 0.575, *p* < 0.0001) and a large effect on anxiety (Cohen’s *d* = 0.733, *p* < 0.0001) (Zainal et al., 2013). Within the veteran population, empirical evidence has shown reduction in symptoms of anxiety, depression, PTSD, and suicidal ideation among veterans after MBSR intervention (Felleman et al., 2016; Kearney et al., 2016; Marchand et al., 2021; Polusny et al., 2015; Serpa et al., 2014).

The promising effects of MBSR on mental health outcomes have led to a growing interest in exploring the underlying mechanisms that help understand the effects. Psychological factors such as self-reported mindfulness, decentering, acceptance, and emotional regulation have been identified as mechanisms of MBSR’s effects (Creswell, 2017). MBSR has been associated with higher levels of self-reported mindfulness, which in turn is associated with decreases in PTSD symptoms among veterans (Polusny et al., 2015). Decentering refers to a process of observing internal experiences from an objective and non-judging stance towards the self (Creswell, 2017; Kessel et al., 2016) and higher levels of decentering after MBSR intervention have been shown to be associated with lower levels of depressive symptoms (Kessel et al., 2016).

Neurobiologically, research suggests that mindfulness interventions may change the function and structure of the brain, which in turn results in improved mental health (Creswell, 2017). MBSR has been shown to increase ventrolateral prefrontal cortex activity, which was associated with reductions in anxiety symptoms (Hölzel et al., 2011). Furthermore Creswell et al. (2016) found that mindfulness meditation increased the resting state functional connectivity of brain networks (the Default Mode Network) and executive control (the Executive Control Network), resulting in improved mental health outcomes. Additionally, MBSR treatment was demonstrated to lead to changes in brain functions associated with a decrease in activation of fear, and stress responses in PTSD patients (Bremner et al., 2017).

The psychological and neurological mechanisms underlying the effect of MBSR on mental health outcomes reflect the paradigm of the mind-body relationship (Kabat-Zinn, 2013) that is informed by the embodied cognition framework. Embodied cognition proposes that the body’s interactions with the environment play a fundamental role in cognitive processing (Barsalou, 2008; Osypiuk et al., 2018). This theory takes a bottom-up approach whereby cognition and emotion are rooted in sensory perceptions and sensory-motor input from specific environments and situations (Borghi and Pecher, 2011; Hauke and Kritikos, 2016; Pietrzak et al., 2018). Consequently, knowledge becomes stored in neural patterns or “simulations” which link bodily states and the original experience with the environment, objects, or people (Barsalou, 1999; Borghi and Pecher, 2011). Therefore, when the same or similar situations to the original (namely, simulations) are present, the feedback loops established from these interactions are activated and influence mood states (Barsalou, 1999; Borghi and Pecher, 2011; Pietrzak et al., 2018).

For veterans suffering from PTSD, these simulations are conditions characterised by intrusive, distressing memories and flashbacks of a traumatic event (American Psychiatric Association [APA], 2013). Psychological and physiological distress is thus triggered by the exposure to internal or external simulations of the traumatic event (APA, 2013), which often leads to persistent avoidant behaviours, negative cognitions, and increasing depressive symptoms (APA, 2013; Felleman et al., 2016). These symptoms may lead to emotional dysregulation in everyday life. Emotional dysregulation is often accompanied by physiological reactions akin to a stress reaction, and the ability to accurately detect and evaluate these embodied signals are fundamental to emotional regulation. This evaluation is followed by developing appropriate regulation strategies that can mitigate and modify the emotional reactions to the stressful event (Price and Hooven, 2018). Interoceptive awareness, which refers to the awareness of bodily signals (Füstös et al., 2013), is essential for emotion regulation. Developing interoceptive skills through mindfulness meditation may aid in reducing PTSD and depressive symptoms by addressing maladaptive coping mechanisms such as thought suppression and emotional numbing (Felleman et al., 2016; Hauke and Kritikos, 2016).

Although research has suggested that MBSR is efficacious for improving veteran mental health, reviews that provide a clear and comprehensive overview of available literature in this field are limited. The search in Cochrane, PROSPERO and eight databases (CINAHL, Emcare, MEDLINE, PsychInfo, PTSDPubs, PubMed, ProQuest Military Database, and SCOPUS) located one systematic review and meta-analysis of the effectiveness of mindfulness-based interventions (MBIs) on military veterans (Goldberg et al., 2020). Goldberg et al.’s (2020) study found that MBIs have a large effect on depression and a medium effect on PTSD at post-treatment. However, there are several limitations in Goldberg et al.’s study. Firstly, the study amalgamated MBSR and Mindfulness Based Cognitive Therapy (MBCT) interventions in the analysis and interpretation, which may obscure the distinct impact of each intervention. Despite the similarities shared by MBSR and MBCT, they represent distinct interventions with distinct foci (Fisher et al., 2023). When multiple MBIs are included in a single systematic review and meta-analysis, the effectiveness of each individual MBI may be masked (Ni et al., 2020). Therefore, evaluating MBSR and MBCT separately is crucial to discern the individual efficacy. Secondly, the employed data analysis methods of Goldberg et al. may have introduced methodological challenges. In Goldberg et al.’s study, the between-group effect was computed by subtracting the within-group effect for the control conditions and comparing that of the MBI conditions. The authors state that this analytic method took account of the potential between-group differences at baseline. However, as argued by Twisk et al. (2018), the between-group differences at baseline can be statistically adjusted, with recommendation to compare the mean scores at post-/follow-up timepoints between intervention and control groups while adjusting for baseline values. Moreover, as pointed out by Bland and Altman (2011), the analytic method of using separate paired tests against baseline and interpreting only one being significant (e.g., the intervention group) as indicating a difference between the intervention and control groups could be “conceptually wrong, statistically invalid, and consequently highly misleading” (p. 6; see Bland and Altman (2011) for more details). Bland and Altman advised that between-group difference should be performed by comparing the differences between the intervention and control groups directly after adjusting the baseline values. Lastly, Goldberg et al.’s study did not investigate the differences between MBIs and other established therapies such as cognitive behavioural therapy (CBT). Evaluating MBIs in relation to alternative therapies is essential for comprehensively understanding their efficacy and positioning in the spectrum of available treatment options. In conclusion, while Goldberg et al.'s (2020) study demonstrated promising results regarding the efficacy of MBIs on depression and PTSD among military veterans, careful consideration of the aforementioned limitations is essential for a nuanced interpretation of the findings and guiding future research in this domain.

The current study aims to address the research gaps in Goldberg et al.’s study by: 1) focusing on the effectiveness of MBSR on depression, PTSD, and mindfulness among military veterans; 2) directly comparing the differences between the treatment and control groups at post-intervention and follow-up timepoints with an adjustment of the baseline values; and 3) comparing the effectiveness of MBSR against other therapies if available. We will also analyse the within-group changes in the single-arm trials and MBSR intervention groups in the RCTs by comparing post-intervention and follow-up against baseline. Although within-group comparison has its limitations and is not generally used as confirmation of the efficacy of an intervention (Evans, 2010), it provides valuable information on how outcomes change in the same group of people after receiving the intervention (MBSR in this case). The following research questions (RQs) are proposed:

RQ1: Are there significant within-group changes in depression, PTSD, and mindfulness, comparing both post-intervention and follow-up against pre-intervention?
RQ2: Are there significant between-group differences in depression, PTSD, and mindfulness between the MBSR intervention and the control groups at post-intervention and follow-up?
RQ3: Are there significant between-group differences in depression and PTSD between MBSR and other therapies at post-intervention and follow-up?

## Method

Guidelines by the Preferred Reporting Items for Systematic Reviews and Meta-Analyses (PRISMA) were followed in the current systematic review and meta-analysis. The current systematic review was registered in PROSPERO (Registration number: CRD42022314834).

### Inclusion and exclusion criteria

Inclusion criteria included clinical trial studies (including single-arm uncontrolled trials and RCTs) published in peer-reviewed journals and registered trials focusing on PTSD and depression in veterans, and mindfulness-based stress reduction (MBSR). The most common mental health conditions veterans may encounter (e.g., anxiety, alcohol/drug misuse and dependence, suicide) were also included because these mental health conditions are often comorbid with PTSD and depression. Exclusion criteria for primary screening included articles without the search terms in the title or abstract, not peer-reviewed, not published in English, review papers, book chapters, thesis submissions, case studies, editorials and letters to the editor, and articles without full-text.

### Search strategy

JN conducted the data search in nine electronic databases between 18th July and 6th August 2021 (CINAHL, Emcare, MEDLINE, PsychInfo, PTSDPubs, PubMed, ProQuest Military Database, Cochrane register, and SCOPUS), for articles published from inception to August 2021. WL repeated the search to confirm the accuracy of the search. The search protocol was performed again on the 20th of December 2022, 7th of October 2023, and 6th of October 2024 to include new articles published since the original search, resulting in additional 281 titles and abstracts for screening and two new articles published in 2022 were included in the full-text methodological quality assessment. Table 1 presents the search strategy in the format of the Cochrane’s PICO search tool (Higgins et al., 2022).

**Table 1.** PICO search strategy.

PICO	Search strategy
Participation	*MeSH term search:* veterans, veterans health, veterans health services, hospitals veterans
	*Keyword search:* former military personnel, former service men, former service women
Intervention	*MeSH term search:* mindfulness, mindfulness-based stress reduction, MBSR, mindfulness-based interventions, meditation, relaxation therapy
Comparison	N/A
Outcome	*MeSH term search:* Mental Health, mental disorders, quality of life
	*Keyword search:* mental health OR quality of life OR mental illness OR mental disorder* OR mental ill health OR suicid* OR substance abuse OR alcohol* OR drug* OR psychos?s OR depress* OR anxiet* OR nervous* OR social anxiet* OR emotional regulat* OR psychological distress OR emotional distress OR trauma OR post-trauma OR PTSD OR affective disorder* OR mood disorder*

### Study selection

Eligible studies were examined by the title and abstract screening, followed by the full-text methodological quality evaluation. The first two authors independently evaluated titles and abstracts of the retrieved articles coded ‘yes’, ‘no’, or maybe’ on blinded excel spreadsheets to determine eligibility (Astridge et al., 2023; Fisher et al., 2023; Li et al., 2024b). The studies marked as a unanimous ‘yes’ were included for further full-text methodological quality assessment. The studies marked unanimously with ‘no’ were excluded. The studies assessed as ‘maybe’ or those in disagreement, were discussed to reach an agreement to include or exclude from the current review (Fisher et al., 2023; Li et al., 2021b).

The full-text methodological quality assessment was performed by all three authors independently to assess the quality of the included studies, using the Mixed Methods Appraisal Tool (MMAT) Version 2018 (Hong et al., 2019). Quality of the studies were determined using the inter-rater agreement measure of Fleiss’ kappa (*k*): *k* < 0.20, 0.40, 0.60 and 0.80 suggesting poor, fair, moderate, substantial, and perfect agreements, respectively (Fleiss, 1971). Articles with *k* lower than 0.40 were discussed to reach an agreement for inclusion or exclusion in the review (Astridge et al., 2023; Fisher et al., 2023; Leow et al., 2024). All included articles had *k*s above 0.40.

### Data extraction

A standard form was used to extract data, which included first author, publishing year, citation, country of the study, sample size, analytic methods, sample population demographics, measures of outcomes and findings (Fisher et al., 2023; Li et al., 2021b). The authors assessed the extracted data by coding the evidence for findings in each article as ‘unequivocal’, ‘credible’ or ‘unsupported’. An evaluation agreement index = ((N_unequivocal_ + N_credible_)/N_reviewers_) for every article was estimated (Astridge et al., 2023; Fisher et al., 2023; Wigg et al., 2024). After the post-rating discussion amongst the authors, all included articles had an agreement index of 1, reaching unanimous agreements.

### Data synthesis

Data synthesis was conducted employing a meta-analysis using the Comprehensive Meta-Analysis (CMA) V3 software (Borenstein et al., 2013). All included studies reported data on several outcome variables, which were based on the same participants. Therefore, the multiple outcomes model was employed to compute the effect sizes of the outcomes, which took correlations between different outcomes in the same study into account (Borenstein et al., 2021). The pooled effect size of an outcome across included studies was estimated using the random effects model.

The within-group comparisons included data of the single-arm uncontrolled trials and the intervention group in RCTs. The mean within-group differences were computed by the means at post-intervention/follow-up deducting the baseline means. For between-group effect analysis, the group differences were computed using the mean scores of an outcome variable at post-intervention and follow-up of the MBSR intervention groups deducting those of the control group after adjusting the baseline values (Fisher et al., 2023).

For studies reporting multiple estimates of effect sizes for an outcome (e.g., multiple effect sizes of subscales for PTSD/mindfulness in the same study), the overall effect size was used in the main meta-analysis to estimate the overall effect size across studies. A two-level meta-analysis was conducted when the overall effect size was not available (Astridge et al., 2023; Fisher et al., 2023; Freedman et al., 2024). Firstly, using the fixed effect model, the multiple effect sizes within each study for an outcome was computed to yield a pooled effect size for the outcome within the study. Secondly, using the random effects model, the pooled effect size obtained from the first step was entered to the main meta-analysis (Hedges, 2019).

Hedges’ *g* was used to report the effect size that was identified as being small, medium, or large as per *g* = 0.20, 0.50, and 0.80, respectively (Cohen, 1988). A default correlation of *r* = 0.50 was used for within-group effects for studies that did not report correlations of the outcome variable between pre-intervention and post-intervention/follow-up (Fisher et al., 2023; White et al., 2019). For the study reporting the standard error (SE) of a mean (Arefnasab et al., 2016), the standard deviation (SD) was obtained from the SE by multiplying by the square root of the sample size (SD = SE × √N; Higgins and Green, 2011).

Heterogeneity was evaluated with *I*^2^ statistics where low, moderate, and high heterogeneity being represented by *I*^2^ = 25, 50, and 75 and over (Borenstein et al., 2019). To investigate publication bias, Egger’s regression test was conducted. Publication bias was identified when *p* values were significant (Borenstein et al., 2019).

### Assessing risk of bias in included studies

An assessment of the risk of bias for each included study was conducted employing the Prediction Model Study Risk of Bias Assessment Tool (PROBAST; Wolff et al., 2019). The overall risks of biases for all included articles were rated as low by the first two authors. Publication bias test was also performed to evaluate if studies with nonsignificant results were withheld from publication.

## Results

**Figure 1.**
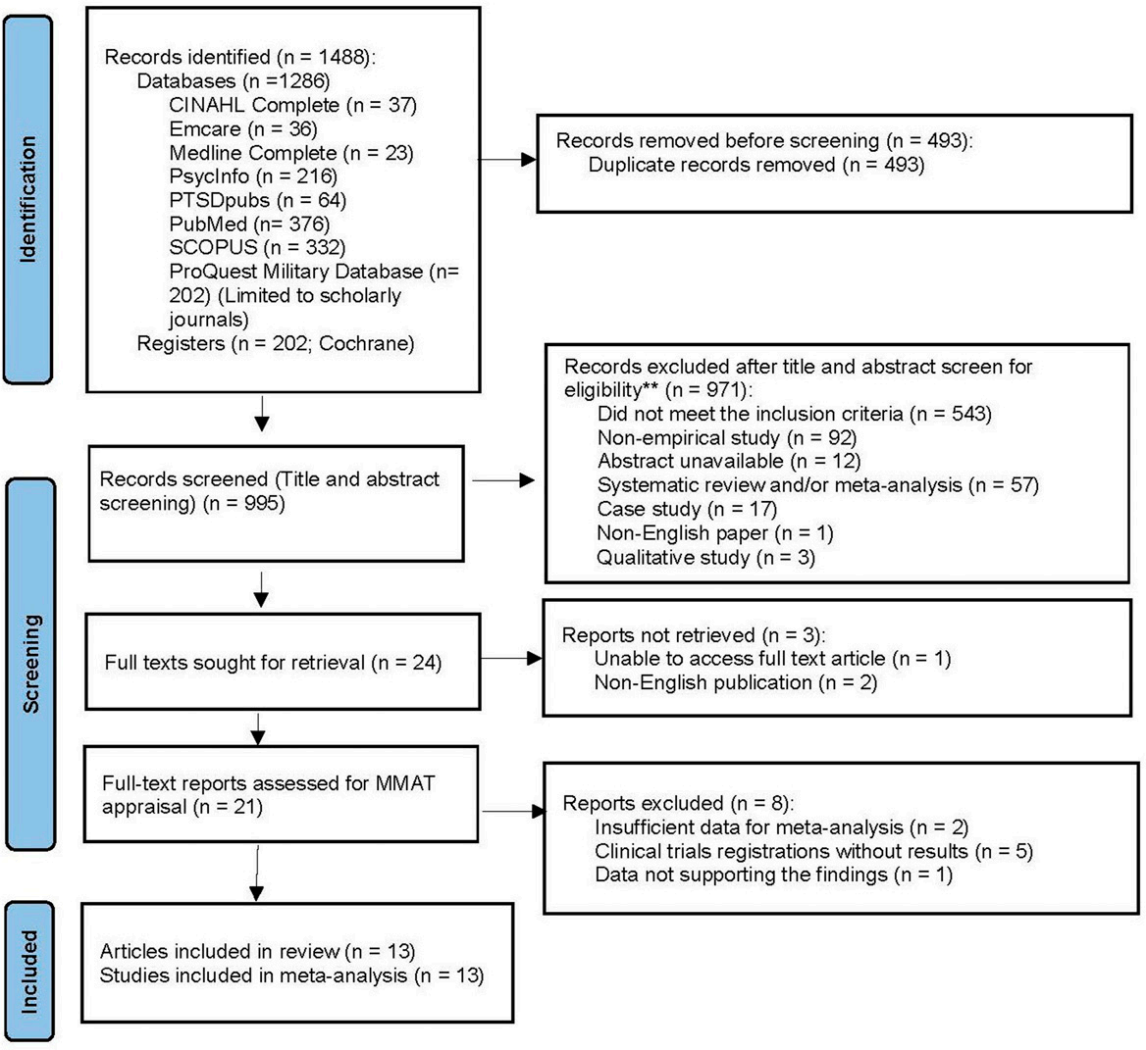
PRISMA flow chart of results of systematic review and meta-analysis.

The PRISMA diagram in Figure 1 shows studies that were included and excluded in the current study. After removing the duplicates, 995 titles were identified in the literature search. Title and abstract screening excluded 971 titles; 24 studies were sought for retrieval for full text screening. The full texts of eight studies were not available. Emails were sent to the corresponding authors of the eight studies to request the full text. Seven full texts were received, including two non-English articles and five clinical trial registrations. The author of one paper did not respond to our request. As a result, three studies were excluded (including the two non-English articles and one full-text unavailable), which left 21 studies for full text analysis. Among the 21 studies, five clinical trial registrations without results were excluded. Two studies did not provide sufficient data for meta-analysis. The authors of these two studies were contacted requesting missing data for meta-analysis, but there was no response. These two studies were thus removed. One study (Saban et al., 2022) was excluded for the following reasons: the authors claimed no significant group differences at baseline in PTSD and depression between MBSR and control groups, however the current research team found significant differences (PTSD: *p* = 0.048, depression: *p* = 0.042). Additionally, the authors claimed participants in the MBSR group reported lowered perceived stress, loneliness, and symptoms of PTSD compared to those in the actively control group, however, the data presented contradicted this. The final number of the included study was 13.

### Characteristics of included studies

Of the 13 studies included in this meta-analysis, the majority were conducted in the USA (*n* = 11), followed by Iran (*n* = 2) with a total of 1131 participants. Eight studies were randomised controlled trials (RCTs) and five were single-arm uncontrolled trials. Participants were predominantly male (80.9%) with an average age of 50.2 years. Most studies (*n* = 11) followed the standardised MBSR protocol (e.g., 2.5 h per week for 8 weeks) with (*n* = 7) or without (*n* = 4) the full-day retreat, while two studies shortened MBSR. Summaries of characteristics and findings of the included studies are presented in Table 2 and Table S1, respectively.

**Table 2.** Characteristics of included studies.

First author	Country	Research design	Demographic information	Psychiatric history	Scales	MBSR protocol
Arch et al. (2013)	USA	Randomised controlled trial (RCT) MBSR vs cognitive behavioural therapy (CBT) with 3 timepoints: Pre- and post-intervention, and 3-month follow up	*N* = 105 Age: M = 45.9 (SD = 13.68) Sex: 83% male, 17% female	Panic disorder: 30.39% Generalised anxiety disorder (GAD): 37.25% Seasonal affective disorder (SAD): 15.69% PTSD: 14.71% Obsessive-compulsive disorder (OCD): 4.90%	Beck Depression Inventory (BDI)	10 weekly × 1.5 h modified MBSR sessions with the full-day retreat
Arefnasab et al. (2016)	Iran	RCT MBSR vs Waiting list with 2 timepoints: Pre- and post-intervention	*N* = 40 Age: M = 50.2 (SD = 5.25) Sex: 100% male	Mental health conditions unreported	General Health Questionnaire (GHQ)	8 weekly × 2.5 h MBSR sessions without the full-day retreat
Bremner et al. (2017)	USA	RCT MBSR vs present-centred group therapy (PCGT) with 3 timepoints: Pre- and post-intervention, and 6-month follow up	*N* = 26 Age: M = 34.0 (SD = 7) Sex: 100% male	Combat-related PTSD	Clinician-administered PTSD Scale (CAPS); Five Facet Mindfulness Questionnaire (FFMQ)	8 weekly × 2.5 h MBSR sessions without the full-day retreat
Davis et al. (2019)	USA	RCT MBSR vs PCGT with 5 timepoints: Pre-, during- (week 3 and week 6) and post-intervention, and 7-week follow up	*N* = 214 Age: M = 51.7 (SD = 10.9) Sex: 80% male, 20% female	PTSD	CAPS; Patient Health Questionnaire-9 (PHQ-9); FFMQ	8 weekly × 1.5 h modified MBSR sessions with the full-day retreat
Felleman et al. (2016)	USA	Single-arm uncontrol trial (SAUT) with 3 timepoints: Pre- and post-intervention, and 4-month follow up	*N* = 116 Age: M = 52.3 (SD = 11.46) Sex: 88% male, 12% female	PTSD	PTSD Checklist Civilian Version (PCL-C); PHQ-9	8 weekly × 2.5 h MBSR sessions with the full-day retreat
Harding et al. (2018)	USA	SAUT with 3 timepoints: Pre- and post-intervention, and 4-month follow up	*N* = 55 Age: M = 52.6 (SD = 11.68) Sex: 85.5% male, 14.5% female	PTSD with comorbid IBS	PCL-C; PHQ-9; FFMQ	8 weekly × 2.5 h MBSR sessions with the full-day retreat
Kearney et al. (2012)	USA	SAUT with 3 timepoints: Pre- and post-intervention, and 4-month follow up	*N* = 92 Age: M = 51.0 (SD = 10.6) Sex: 76% male, 24% female	Depression: 58.7% Bipolar disorders: 7.6% General anxiety: 17.4% PTSD: 34.8%	PCL-C; PHQ-9; FFMQ	8 weekly × 2.5h MBSR sessions with the full-day retreat
Kearney et al. (2013)	USA	RCT MBSR vs treatment as usual (TAU) with 3 timepoints: Pre- and post-intervention, and 4-month follow up	*N* = 47 Age: M = 52.0 (SD = 13.4) Sex: 78% male, 22% female	PTSD	PCL-C; PHQ-9	8 weekly × 2.5 h MBSR sessions with the full-day retreat
Kearney et al. (2016)	USA	RCT MBSR vs treatment as usual (TAU) with 3 timepoints: Pre and post-intervention, and 6-month follow up	*N* = 55 Age: M = 51.3 (SD = 6.8) Sex: 85.5% male, 14.5% female	Depression: 50% Anxiety: 19.2% PTSD: 29.8%	PCL-C; PHQ-9; FFMQ	8 weekly × 2.5 h MBSR sessions with the full-day retreat
Kluepfel et al. (2013)	USA	SAUT with 2 timepoints: Pre- and post-intervention	*N* = 30 Age: M = 60.0 (SD = 11.45) Sex: 84% male, 16% female	PTSD: 82.1% Depression: 39.2% Bipolar disorder: 10.7% Personality Disorder: 14.2% Other anxiety disorder: 10.7%	BDI; Mindfulness Attention Awareness Scale (MAAS)	8 weekly × 2.5 h MBSR sessions with the full-day retreat
Omidi et al. (2013)	Iran	RCT MBSR vs TAU with 2 timepoints: Pre and post-intervention	*N* = 62 Age: M = 35.5 (SD unreported) Sex: 100% male	PTSD	Inventory of Mood States (IMS)	8 weekly × 2.5 h MBSR sessions without the full-day retreat
Serpa et al. (2014)	USA	SAUT with 2 timepoints: Pre- and post-intervention	*N* = 79 Age: M = 60.0 (SD = 7) Sex: 89% male, 11% female	Mental health conditions unreported	PHQ-9; FFMQ	8 weekly × 2.5 h MBSR sessions without the full-day retreat
Shapira et al. (2022)	USA	RCT MBSR vs PCTG with 3 timepoints: pre-, post- and 4-month follow up	*N* = 210 Age: M = 55.0 (SD = 12) Sex: 84% male, 16% female	PTSD	PHQ-9, FFMQ	8 weekly × 2.5 h MBSR sessions with the full-day retreat

### Test of RQ1

#### Within-group effects comparing post-intervention against pre-intervention

**Figure 2.**
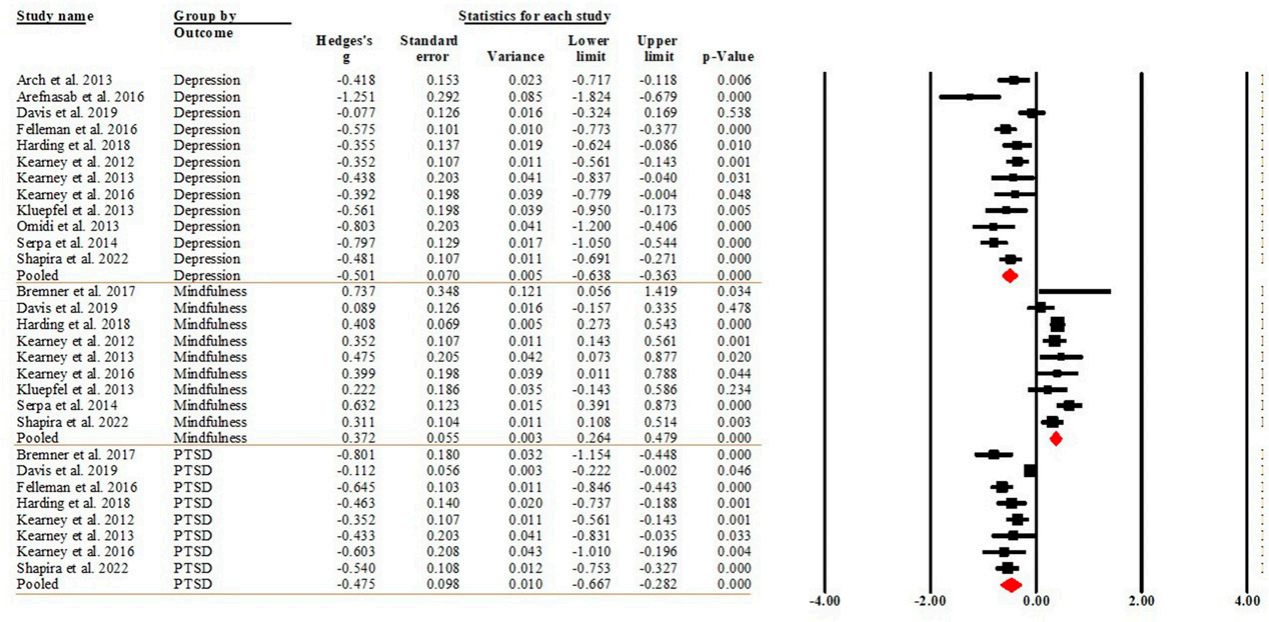
Forest plot of the within-group effects comparing post-intervention against pre-intervention.

Of the 13 studies, 12 reported within-group differences in depression (Arch et al., 2013; Arefnasab et al., 2016; Davis et al., 2019; Felleman et al., 2016; Harding et al., 2018; Kearney et al., 2012, 2013, 2016; Kluepfel et al., 2013; Omidi et al., 2013; Serpa et al., 2014; Shapira et al., 2022), 8 reported within group differences for PTSD comparing the post-intervention against the baseline (pre-intervention) (Bremner et al., 2017; Davis et al., 2019; Felleman et al., 2016; Harding et al., 2018; Kearney et al., 2012, 2013, 2016; Shapira et al., 2022), and 9 reported within-group differences for mindfulness (Bremner et al., 2017; Davis et al., 2019; Harding et al., 2018; Kearney et al., 2012, 2013, 2016; Kluepfel et al., 2013; Serpa et al., 2014; Shapira et al., 2022). The pooled effect sizes indicated that MBSR had medium effect sizes on depression (Hedge’s *g* = −0.501, 95%CI [−0.638, −0.363], *p* < 0.001) and PTSD (Hedge’s *g* = −0.475, 95%CI [−0.667, −0.282], *p* < 0.001), and a small effect size on mindfulness (Hedge’s *g* = 0.372, 95%CI [0.264, 0.479], *p* < 0.001).That said, MBSR significantly reduced depression and PTSD by about 0.501 and 0.474 standard deviations (SDs), respectively; and significantly increased mindfulness by 0.372 SDs. Figure 2 displays the forest plot of the analysis. The *I*^2^ = 62.815 (*p* = 0.002) for depression and *I*^2^ = 81.525 (*p* < 0.001) for PTSD indicated that heterogeneity was moderate and substantial, respectively. The *I*^2^ = 34.577 (*p* = 0.141) for mindfulness indicated that heterogeneity was low. The Egger’s regression test (intercept = −2.227, *t* = 1.357, *df* = 27; *p* = 0.186) suggested publication bias was not detected.

Meta-regression was performed to determine which factors contributed to the heterogeneity. The moderators of sample size (Q = 0.22, *df* = 1, *p* = 0.619), publishing year (Q = 0.30, *df* = 1, *p* = 0.586) and MBSR protocol (Q = 0.98, *df* = 2, *p* = 0.613) were not accountable for the substantial heterogeneity. The moderators of measure (Q = 93.38, *df* = 8, *p* < 0.001) and country (Q = 5.84, *df* = 1, *p* = 0.016), were found to be responsible for the heterogeneity.

#### Within-group effects comparing follow-up against pre-intervention

**Figure 3.**
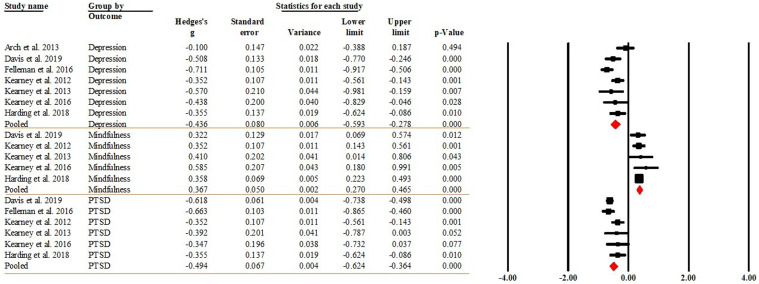
Forest plot of the within-group effects comparing follow-up against pre-intervention.

Seven studies reported within-group differences in depression (Arch et al., 2013; Davis et al., 2019; Felleman et al., 2016; Harding et al., 2018; Kearney et al., 2012, 2013, 2016), six reported within-group differences for PTSD (Davis et al., 2019; Felleman et al., 2016; Harding et al., 2018; Kearney et al., 2012, 2013, 2016), and five reported within-group differences for mindfulness (Davis et al., 2019; Harding et al., 2018; Kearney et al., 2012, 2013, 2016) at follow-up against the pre-intervention timepoints. The pooled effect sizes indicated that MBSR had small effect sizes on depression (Hedge’s *g* = −0.436, 95%CI [−0.593, −0.278], *p* < 0.001), PTSD (Hedge’s *g* = −0.494, 95%CI [−0.624, −0.364], *p* < 0.001), and mindfulness (Hedge’s *g* = 0.367, 95% CI [0.270, 0.465], *p* < 0.001). That is, MBSR significantly reduced depression and PTSD by about 0.436 and 0.494 SDs, respectively; and increased mindfulness by 0.367 SDs. Figure 3 displays the forest plot of the analysis. The *I*^2^ = 56.338 (*p* = 0.033) for depression, *I*^2^ = 47.246 (*p* = 0.091) for PTSD, and *I*^2^ = zero (*p* = 0.858) for mindfulness indicated that heterogeneity was moderate, low and trivial, respectively. The Egger’s regression test (intercept = 0.801, *t* = 0.325, df = 16, *p* = 0.749) suggested publication bias was not detected.

Meta-regression was performed to determine which factors contributed to the heterogeneity. The moderators of sample size (Q = 0.53, *df* = 1, *p* = 0.468), publishing year (Q = 0.11, *df* = 1, *p* = 0.744) and MBSR protocol (Q = 0.01, *df* = 1, *p* = 0.914) were not predictors for the heterogeneity. The moderator of measure (Q = 16.42, *df* = 5, *p* = 0.006) was accountable for the significant heterogeneity.

#### Sensitivity analysis

Two sensitivity analyses were performed to examine whether the results were robust to the decisions made in the process of including the modified MBSR in the meta-analysis. Two studies utilising modified MBSR (Arch et al., 2013; Davis et al., 2019) in the within-group analysis (comparing post-intervention and follow-up against pre-intervention) were removed. The results showed that the significance and direction of the effects on all outcomes did not change (Tables S2 and S3).

### Test of RQ2

#### Effects between MBSR and control groups at post-intervention

**Figure 4.**
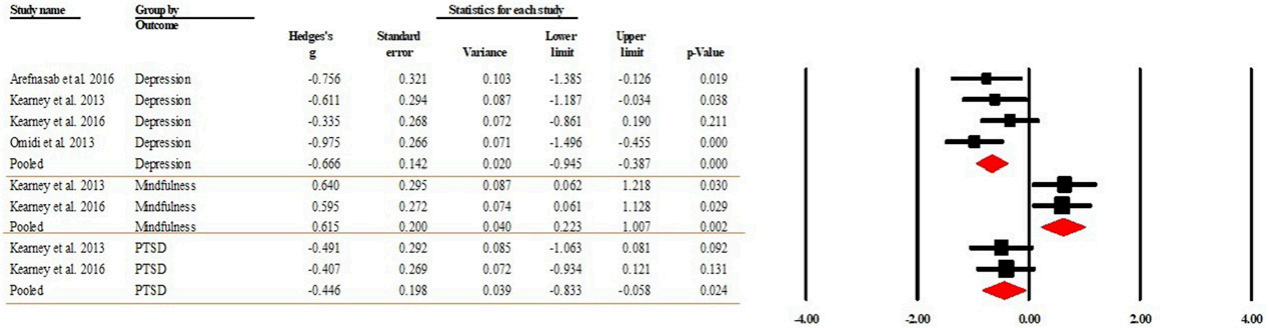
Forest plot of the effects between MBSR and control groups at post-intervention.

Of the 13 included studies, four, two and two studies reported between-group differences between the MBSR and control (TAU or waiting list [WL]) groups at the post-intervention timepoint in depression (Arefnasab et al., 2016; Kearney et al., 2013, 2016; Omidi et al., 2013), PTSD (2019; Kearney et al., 2013; Kearney et al., 2016), and mindfulness (Kearney et al., 2013, 2016), respectively. The pooled effect sizes indicated that MBSR had medium effect sizes on depression (Hedge’s *g* = −0.666, 95% CI [−0.945, −0.387], *p* < 0.001), PTSD (Hedge’s *g* = −0.446, 95% CI [−0.833, −0.058], *p* = 0.024), and mindfulness (Hedge’s g = 0.615, 95% CI [0.223, 1.007], *p* = 0.002). That said, depression and PTSD significantly decreased by 0.446 and 0.615 SDs, respectively, in the MBSR compared to control/TAU groups; and mindfulness significantly increased by 0.615 SDs in the MBSR group. Figure 4 presents the forest plot of the analysis. The *I*^2^ was zero for all three outcomes with *p* = 0.393 for depression, *p* = 0.911 for PTSD and *p* = 0.831 for mindfulness, indicating that heterogeneity was trivial. Meta-regression thus was not performed for moderator analysis. The Egger’s regression test (intercept = −1.228, *t* = 0.093, *df* = 6; *p* = 0.929) suggested publication bias was not detected.

#### Effects between MBSR and control groups at follow-up

**Figure 5.**
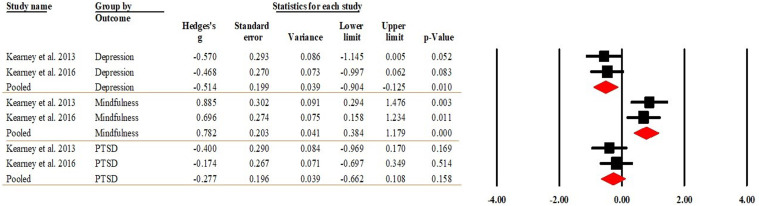
Forest plot of the effects between MBSR and control groups at follow-up.

Two studies each (Kearney et al., 2013, 2016) reported the differences between the MBSR and control (TAU and WL) groups at follow-up in depression and PTSD. Two studies (Kearney et al., 2013, 2016) reported mindfulness. The pooled effect sizes indicated that MBSR had a medium effect size on depression (Hedge’s *g* = −0.514, 95% CI [−0.904, −0.125], *p* = 0.010) and mindfulness (Hedge’s *g* = 0.782, 95% CI [0.384, 1.179], *p* < 0.001). The pooled effect size showed that MBSR had no effect on PTSD (Hedge’s *g* = −0.277, 95% CI [−0.662, 0.108], *p* = 0.158). That said, depression significantly decreased by 0.514 SDs in the MBSR compared to control/TAU groups; and mindfulness significantly increased by 0.782 SDs in the MBSR group. Figure 5 displays the forest plot of the analysis. The heterogeneity indicator *I*^2^ was zero for all three outcomes, with *p* being 0.798 for depression, 0.568 for PTSD and 0.642 for mindfulness, suggesting that heterogeneity was trivial. The Egger’s regression test (intercept = 9.500, *t* = 0.444, *df* = 4, *p* = 0.680) suggested publication bias was not detected. Due to the small number of studies, sensitivity analysis was not performed.

### Test of RQ3

#### Effects between MBSR and other therapies at post-intervention

**Figure 6.**
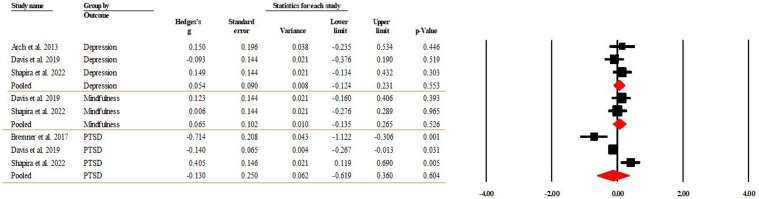
Forest plot of the effects between MBSR and CBT/PCGT groups at post-intervention.

Among the included studies, there was one RCT compared MBSR with CBT (Arch et al., 2013) and three compared MBSR to present-centred group therapy (PCGT) (Bremner et al., 2017; Davis et al., 2019; Shapira et al., 2022). Three studies each reported the between-group differences at the post-intervention timepoint in depression (Arch et al., 2013; Davis et al., 2019) and PTSD (Bremner et al., 2017; Davis et al., 2019). Two studies reported mindfulness (Davis et al., 2019; Shapira et al., 2022). The pooled effect sizes indicated that depression (Hedge’s *g* = 0.054, 95% CI [−0.124, 0.231], *p* = 0.553), PTSD (Hedge’s *g* = −0.130, 95% CI [−0.619, 0.360], *p* = 0.604), and mindfulness (Hedge’s *g* = 0.065, 95% CI [−0.135, 0.265], *p* = 0.526) were at similar levels between the MBSR and CBT/PCGT groups. Figure 6 present the forest plot of the analysis. The *I*^2^ = zero for both depression (*p* = 0.426) and mindfulness (*p* = 0.566) indicated that heterogeneity was trivial. The *I*^2^ = 90.521 (*p* < 0.001) for PTSD indicated that heterogeneity was high. Due to the low number of studies included in the analysis, mete-regression for moderator analysis was not performed. The Egger’s regression test (intercept = 1.081, *t* = 0.573 *df* = 6; *p* = 0.587) suggested publication bias was not detected.

#### Effects between MBSR and CBT/PCGT groups at follow-up

**Figure 7.**
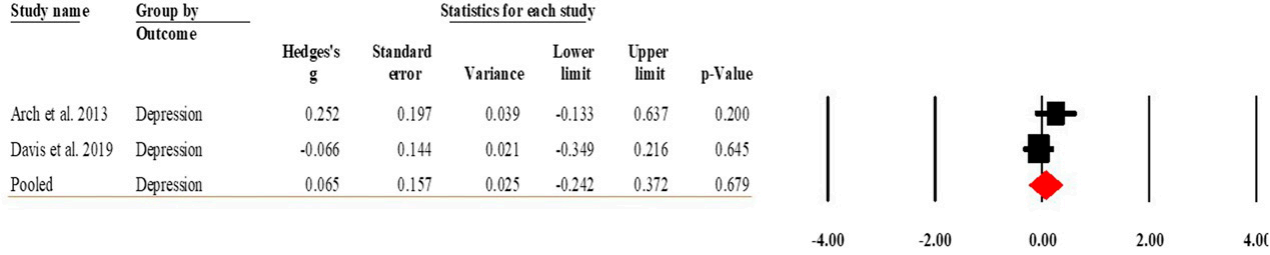
Forest plot of the effects between MBSR and CBT/PCGT groups at follow-up.

One study reported the differences at the follow-up timepoint in depression comparing MBSR to CBT (Arch et al., 2013) and one compared MBSR to PCGT (Davis et al., 2019). The pooled effect sizes indicated that the levels of depression were similar in the MBSR and CBT/PCGT groups (Hedge’s *g* = 0.065, 95% CI [−0.242, 0.372], *p* = 0.679). Figure 7 present the forest plot of the analysis. The *I*^2^ = 41.427 (*p* = 0.191) for depression indicated that heterogeneity was low. Due to the low number of studies included in the analysis, publication bias and sensitivity analyses were not performed.

## Discussion

To the authors’ knowledge, this is the first systematic review and meta-analysis to identify the effectiveness of MBSR in reducing depression and PTSD symptoms and improving mindfulness in military veterans. A total of 13 studies with 1131 participants were included in the systematic review and meta-analysis.

Our findings from both within- and between-group comparisons suggest that MBSR demonstrated small to medium effects in reducing depressive and PTSD symptoms and improving mindfulness in veterans at post-intervention. These effects endured and remained consistent in the within-group comparison at follow-up (Range: 2-6 months; Mean = 3.8 months). However, when comparing MBSR to control groups at follow-up, the effects on depression and mindfulness persisted, but not on PTSD. Our findings are consistent with the findings in Goldberg et al.’s (2020) systematic review on the efficacy of MBIs for military veterans, which also found that MBIs are superior to the control group on measures of depression and PTSD at post-treatment. The authors additionally found that at follow-up (mean length = 3.19 months), MBIs continued to outperform the control group on reducing depression, but not PTSD.

Informed by the embodied cognition framework, the body-mind relationship paradigm suggests that the signals from the body concerning both internal and external stressors encountered during military service and in their everyday life can significantly impact cognitions, motivation, and mood states (Hauke and Kritikos, 2016; Osypiuk et al., 2018). This influence may contribute to the manifestation of symptoms associated with depression and PTSD. Through MBSR practice, which increases mindfulness and encourages participants to acknowledge challenging and complicated body and mind relationships without judgement and avoidance, veterans may be able to adopt a holistic approach. This approach assists them to develop strategies to regulate emotional and behavioural responses to the stressors (Schure et al., 2018). Consequently, with the increased levels of mindfulness veterans may obtain a better sense of control in stressful situations which may result in reduced physiological reactivity and symptoms of PTSD and depression (Chiesa et al., 2014; Priya and Kalra, 2018; Schure et al., 2018).

Several factors may contribute to the insignificant result in PTSD comparing MBSR and control groups at follow-up. Firstly, only two studies were included in the meta-analysis. Such a small volume of data may decrease statistical power to detect effect sizes. Secondly, the complexity of PTSD in veterans may contribute to the insignificant treatment gain at follow-up. This finding indicates that MBSR alone may not be a viable replacement for the recommended trauma-focused therapies for treating veteran PTSD (e.g., prolonged exposure therapy, cognitive processing therapy, and trauma-focused cognitive behavioural therapy). Instead, MBSR may serve as an important foundation treatment that enhances patients’ motivation, willingness, and ability to engage with the full-length trauma-focused treatments for better treatment outcomes (Possemato et al., 2016). MBSR as a stress reduction adjunct may reduce dropout and enhance ongoing engagement for veterans to complete trauma-focused therapies.

Our comparison between MBSR and CBT/PCGT indicates that there were no significant differences between MBSR and CBT/PCGT with regards to the outcomes of depression, PTSD and mindfulness, meaning that MBSR is as effective as CBT and PCGT. This finding is consistent with findings in recent reviews that compare the efficacy between MBSR and CBT in chronic pain (Khoo et al., 2019) and mental health outcomes of anxiety, depression and sleep quality (Li et al., 2021a). These two reviews found no significant differences between MBSR and CBT in terms of the treatment outcomes of chronic pain, anxiety, depression, and sleep quality. Our findings indicate that MBSR could be used as an alternative psychotherapy to CBT/PCGT for reducing veteran depressive and PTSD symptoms.

The findings of the sensitivity analysis indicate that removing studies using shortened MBSR did not alter the significance or direction of the effects on the outcomes. This finding is consistent with the finding in a recent meta-analysis that found that the efficacy of MBSR in diabetes patients was not moderated by the MBSR protocols (e.g., eight-week MBSR with/without full-day retreat and shortened MBSR) (Fisher et al., 2023). Future RCTs are warranted to evaluate the effect of low-dose MBSR on the mental health of veterans.

Results from the heterogeneity analyses indicate the heterogeneities in depression and PTSD in the pre-post within-group comparisons; and in PTSD between the MBSR and CBT/PCGT groups at post intervention were substantial. The meta-regression analyses show that scales and countries, where the studies were conducted, contributed to the heterogeneities. The high levels of heterogeneity suggest the effects of MBSR on the outcome variables are low in some veteran populations and high in others (Borenstein, 2019). Therefore, generalising results from the current study will be taken with caution.

There are several limitations worth noting within the current study. First, the limited number of RCTs (Arch et al., 2013; Arefnasab et al., 2013; Bremner et al., 2017; Davis et al., 2019; Felleman et al., 2016; Kearney et al., 2013, 2016; Omidi et al., 2013) in the meta-analysis is likely to reduce the statistical power for detecting differences between the MBSR and control groups. Second, the underrepresentation of females in the included studies (Arch et al., 2013; Arefnasab et al., 2013, 2016; Harding et al., 2018; Kearney et al., 2013, 2016) hinders a comprehensive understanding of MBSR’s effects in diverse populations. This is consistent with a previous review of MBIs with veteran populations where participants were 85% male across all studies (Marchand et al., 2021). Third, the geographical representation being restricted to the USA and Iran, limits the generalisability of the findings to all veteran populations.

Despite the limitations, the current study offers important clinical implications. The positive impact of MBSR on veterans’ mental health can enhance their engagement with other treatments, especially trauma-focussed ones. Treatment avoidance and attrition are well documented challenges within veteran populations, both through a treatment lens (Sciarrino et al., 2022; Steenkamp et al., 2020) and through the lens of PTSD psychopathology (APA, 2013). Mindfulness practice as a mild form of exposure therapy (Baer, 2003; Felleman et al., 2016; Kearney et al., 2012) opens veterans to confronting uncomfortable experiences (emotional, environmental, social) through increased acceptance, non-judgement, and heightened awareness of the present moment (Felleman et al., 2016; Hauke and Kritikos, 2016; Kabat-Zinn, 2003, 2013; Omidi et al., 2013; Schure et al., 2018). Consequently, the automatisation of avoidance behaviours triggered by trauma-based treatments could potentially be mitigated through the development of a ‘mindful mind’ (Kabat-Zinn, 2005). This mental state may provide new neural simulations (Barsalou, 1999; Borghi and Pecher, 2011) that support the cognitive system to interpret the treatment situations as safe (Balcetis and Cole, 2009; Tuerk et al., 2011).

## Conclusion

Findings from this review and meta-analysis indicate MBSR is moderately effective in reducing depression and PTSD symptoms and improving mindfulness in military veterans. These results suggest that MBSR could be utilised as a non-trauma focused therapy for improving veterans’ mental health.

## Supplemental Material

**Supplemental Material -** The effectiveness of mindfulness-based stress reduction (MBSR) on depression, PTSD, and mindfulness among military veterans: A systematic review and meta-analysis Supplemental Material for The effectiveness of mindfulness-based stress reduction (MBSR) on depression, PTSD, and mindfulness among military veterans: A systematic review and meta-analysis by Wendy Wen Li, Jaime Nannestad, Timothy Leow and Carolyn Heward in Health Psychology Open.
