# Supplementary material for: The effectiveness of mindfulness-based stress reduction (MBSR) on depression, PTSD, and mindfulness among military veterans: A systematic review and meta-analysis
Source: Health Psychol Open. 2024 Nov 21;11:20551029241302969. doi: 10.1177/20551029241302969 (PMC11583271; doi:10.1177/20551029241302969)
Supplement: Supplemental Material - The effectiveness of mindfulness-based stress reduction (MBSR) on depression, PTSD, and mindfulness among military veterans: A systematic review and meta-analysis [file sj-pdf-1-hpo-10.1177_20551029241302969.pdf]

Table S1. *Summary of the findings of the included study*

Author & year	Within-group means at different timepoints									Between-group mean differences at post and follow-up timepoints					
	PTSD			Depression			Mindfulness			PTSD		Depression		Mindfulness	
	Pre-test	Post-test	Follow-up	Pre-test	Post-test	Follow-up	Pre-test	Post-test	Follow-up	Post-test	Follow-up	Post-test	Follow-up	Post-test	Follow-up
Arch (2013)				25.97 (11.61)	21.36 (15.26)	24.53 (15.68)									
Arefnasab (2016)				9.44 (1.01)	5.77 (0.81)										
Bremner (2017) - T	56 (29)	28 (20)					121 (11)	139 (22)		15				-12	
Bremner (2017) - I	10 (9)	3 (4)								5					
Bremner (2017) - Av	16 (9)	8 (7)								0					
Bremner (2017) - Ar	31 (14)	16 (11)								11					
Davis (2019) - T	84.3 (19.5)	59.1 (27.1)	64.8 (30.8)	27.7 (6.7)	23.8 (7.6)	24 (7.6)	109.9 (21)	116.9 (20.2)	117.2 (23.6)	5.4	-1.9	0.7	0.5	-2.3	-1.1
Davis (2019) - B	23.4 (8.2)	14.8 (10.3)	17 (10.8)							1.7	-0.2				
Davis (2019) - C	34.6 (8.4)	24.9 (12.5)	26.5 (13.8)							0.8	-1.2				
Davis (2019) - D	26.3 (6.3)	19.5 (7.8)	21.3 (8.9)							2.8	-0.5				
Davis (2019) - PCL	63.1 (12.2)	53.8 (17.4)	56.2 (16.5)							-1.2	-2.4				
Felleman (2016)	59.2 (10.8)	51 (13.9)	50.4 (14.6)	15.7 (5.8)	12.2 (6.2)	11.2 (6.5)									
Harding (2018)	Mean Difference Post: -7.63 (11.18) p < 0.001 FU: -7.03 (12.46) p < 0.01			Mean Difference Post: -3.27 (6.09) p < 0.01 FU: -3.05 (5.28) p < 0.01											

Harding (2018) - D							Mean Difference<br>Post: 3.02 (5.35) p < 0.001<br>FU: 2.70 (6.74) p < 0.05								
Harding (2018) - A							Mean Difference<br>Post: 2.59 (7.24) p < 0.01<br>FU: 4.40 (5.77) p < 0.001								
Harding (2018) - NJ							Mean Difference<br>Post: 4.00 (7.98) p < 0.01<br>FU: 4.00 (7.33) p < 0.01								
Harding (2018) - NR							Mean Difference<br>Post: 3.35 (5.88) p < 0.001<br>FU: 2.85 (6.24) p < 0.01								
Kearney (2012)	52.4 (16.3)	43.4 (16.3)	41.9 (16.8)	14.7 (6.7)	11 (7.2)	9.8 (7.3)	108 (25.3)	126.2 (27.7)	127.9 (127.9)						
Kearney (2013)	59.8 (11)	52.4 (13)	54.4 (15)	15.9 (6)	12 (6)	12.3 (6)	105.9 (20)	115.3 (18)	114.0 (2)	6.05	5.73	3.45	3.22	-13.67	-14.82
Kearney (2016)	29 (11.2)	20.7 (9.1)	21.7 (10.8)	14.4 (5.5)	10.2	9.5	115.8	126	129.4	4	1.9	2.2	2.8	-11.5	-14.8
Kluepfel (2013)				25.78	17.68		41.68	46.68							
Omidi (2013)				89.3	72							20.2			
Serpa (2014)				11.85	8.13		120.71	131.44							

Table S2. *Sensitivity analysis comparing pre-test to post-test in within-group difference (Removing the shortened MBSR studies)*

Model	Study name	Group by	Statistics for each study					
			Hedges's g	SE	Variance	Lower limit	Upper limit	p-Value
	Arefnasab et al. 2016	Depression	-1.251	0.292	0.085	-1.824	-0.679	0.000
	Felleman et al. 2016	Depression	-0.575	0.101	0.010	-0.773	-0.377	0.000
	Harding et al. 2018	Depression	-0.355	0.137	0.019	-0.624	-0.086	0.010
	Kearney et al. 2012	Depression	-0.352	0.107	0.011	-0.561	-0.143	0.001
	Kearney et al. 2013	Depression	-0.438	0.203	0.041	-0.837	-0.040	0.031
	Kearney et al. 2016	Depression	-0.392	0.198	0.039	-0.779	-0.004	0.048
	Kluepfel et al. 2013	Depression	-0.561	0.198	0.039	-0.950	-0.173	0.005
	Omidi et al. 2013	Depression	-0.803	0.203	0.041	-1.200	-0.406	0.000
	Serpa et al. 2014	Depression	-0.797	0.129	0.017	-1.050	-0.544	0.000
	Shapira et al. 2022	Depression	-0.481	0.107	0.011	-0.691	-0.271	0.000
Random		Depression	-0.553	0.067	0.004	-0.684	-0.422	0.000
	Bremner et al. 2017	Mindfulness	0.737	0.348	0.121	0.056	1.419	0.034
	Harding et al. 2018	Mindfulness	0.408	0.069	0.005	0.273	0.543	0.000
	Kearney et al. 2012	Mindfulness	0.352	0.107	0.011	0.143	0.561	0.001
	Kearney et al. 2013	Mindfulness	0.475	0.205	0.042	0.073	0.877	0.020
	Kearney et al. 2016	Mindfulness	0.399	0.198	0.039	0.011	0.788	0.044
	Kluepfel et al. 2013	Mindfulness	0.222	0.186	0.035	-0.143	0.586	0.234
	Serpa et al. 2014	Mindfulness	0.632	0.123	0.015	0.391	0.873	0.000
	Shapira et al. 2022	Mindfulness	0.311	0.104	0.011	0.108	0.514	0.003
Random		Mindfulness	0.407	0.043	0.002	0.323	0.491	0.000
	Bremner et al. 2017	PTSD	-0.801	0.180	0.032	-1.154	-0.448	0.000
	Felleman et al. 2016	PTSD	-0.645	0.103	0.011	-0.846	-0.443	0.000
	Harding et al. 2018	PTSD	-0.463	0.140	0.020	-0.737	-0.188	0.001
	Kearney et al. 2012	PTSD	-0.352	0.107	0.011	-0.561	-0.143	0.001
	Kearney et al. 2013	PTSD	-0.433	0.203	0.041	-0.831	-0.035	0.033
	Kearney et al. 2016	PTSD	-0.603	0.208	0.043	-1.010	-0.196	0.004
	Shapira et al. 2022	PTSD	-0.540	0.108	0.012	-0.753	-0.327	0.000
Random		PTSD	-0.532	0.055	0.003	-0.640	-0.425	0.000

Table S3. *Sensitivity analysis comparing pre-test to follow up in within-group difference (Removing the shortened MBSR studies)*

Model	Group by	Study name	Statistics for each study					
			Hedges's g	SE	Variance	Lower limit	Upper limit	p-Value
	Depression	Felleman et al. 2016	-0.711	0.105	0.011	-0.917	-0.506	0.000
	Depression	Kearney et al. 2012	-0.352	0.107	0.011	-0.561	-0.143	0.001
	Depression	Kearney et al. 2013	-0.570	0.210	0.044	-0.981	-0.159	0.007
	Depression	Kearney et al. 2016	-0.438	0.200	0.040	-0.829	-0.046	0.028
	Depression	Harding et al. 2018	-0.355	0.137	0.019	-0.624	-0.086	0.010
Random	Depression		-0.491	0.070	0.005	-0.628	-0.353	0.000
	Mindfulness	Kearney et al. 2012	0.352	0.107	0.011	0.143	0.561	0.001
	Mindfulness	Kearney et al. 2013	0.410	0.202	0.041	0.014	0.806	0.043
	Mindfulness	Kearney et al. 2016	0.585	0.207	0.043	0.180	0.991	0.005
	Mindfulness	Harding et al. 2018	0.358	0.069	0.005	0.223	0.493	0.000
Random	Mindfulness		0.385	0.071	0.005	0.246	0.525	0.000
	PTSD	Felleman et al. 2016	-0.663	0.103	0.011	-0.865	-0.460	0.000
	PTSD	Kearney et al. 2012	-0.352	0.107	0.011	-0.561	-0.143	0.001
	PTSD	Kearney et al. 2013	-0.392	0.201	0.041	-0.787	0.003	0.052
	PTSD	Kearney et al. 2016	-0.347	0.196	0.038	-0.732	0.037	0.077
	PTSD	Harding et al. 2018	-0.355	0.137	0.019	-0.624	-0.086	0.010
Random	PTSD		-0.449	0.070	0.005	-0.586	-0.313	0.000
